# Understanding rural household behavior: Beyond Boserup and Becker

**DOI:** 10.1111/agec.12540

**Published:** 2019-11-27

**Authors:** Cheryl R. Doss, Agnes R. Quisumbing

**Affiliations:** ^1^ Oxford Department of International Development University of Oxford Oxford UK; ^2^ International Food Policy Research Institute, IFPRI Washington, DC USA

**Keywords:** agricultural household, gender, household, intrahousehold, rural development, D1, D13, Q12

## Abstract

New data and new methods have provided many new insights into rural households in the past 50 years. We analyze what we have learned from household models since Boserup and Becker, using this to frame more recent findings about household behavior from three types of studies: observational studies, experimental games, and impact evaluations. More sex‐disaggregated data, as well as data that are collected at smaller units, such as agricultural plots, have allowed us to better understand agricultural productivity, risk sharing, and spousal cooperation. However, the focus on bargaining within households has often led us to ignore the cooperation that occurs within households. Many resources are owned and managed jointly by household members and many decisions are made jointly, although not all parties necessarily have equal voice in these decisions. Research demonstrating that households often do not reach efficient outcomes suggests that we still have much to learn about rural household behavior. Understanding both individual roles within households and the levels of cooperation, including joint decision making and ownership of resources, is essential to analysis of households, especially in rural areas where households engage in both production and consumption.

## 1 INTRODUCTION

Theoretical and empirical research on rural households has shaped the way economists think about household behavior and gender dynamics over the last 50 years. Boserup's (1970) *Women's Role in Economic Development* and Becker's (1981) *Treatise on the Family*, published a decade apart, have influenced approaches both to understanding rural households and designing agricultural development policies and programs. Based on her analysis of data from Africa and Asia, Boserup (1970) hypothesized that in areas with low population density, women, rather than men, do more farm work, whereas in densely populated areas, agriculture is intensive in male labor. In her view, men also tended to apply modern scientific methods to cultivate cash crops, while women continued to produce food crops using traditional methods. Although the strict gender divisions of labor in “male” or “female” farming systems and the specialization of women and men in food crops and cash crops, respectively, have been challenged by recent evidence (see, e.g., Doss, 2002), the idea of “men's crops” and “women's crops” on plots that men and women cultivate solely has endured.

Another persistent paradigm is Becker's unitary model of the household (1981), which assumed either that all household members have the same preferences, pool all resources, and agree on all decisions, or that one household member makes the decisions for everyone. Becker takes social norms on gender roles as exogenous, with men specializing in production activities and women specializing in reproduction activities. This model has been challenged, both theoretically and empirically, by collective models of household behavior that allow decision makers to have different preferences and do not assume a single household welfare index or utility function (Chiappori, 1992).

The challenges to Boserup and Becker arose from the growth of empirical literature on household decision making in developing countries, stimulated by theoretical developments in modeling household behavior and intrahousehold allocation and by the increased availability of sex‐disaggregated data on labor, asset ownership, and decision making with which to test these household models. Early work that tested the unitary model of the household against the collective model—often using a bargaining framework—frequently rejected the assumptions of the unitary model.

New innovations in data collection at the plot level often included information on the plot owner or manager. This allows for analysis of agricultural productivity at the individual level, rather than only at the household level. Sex‐disaggregated data on income and assets facilitate analysis of how shocks affected household members differently. In addition, some surveys have begun to interview multiple people within a single agricultural household, allowing analyses of the responses of different household members. Research using lab‐in‐the‐field experiments has provided a means to explicitly test the extent to which household members cooperate. These results suggest that households do not necessarily take advantage of all of the opportunities to increase total household income, albeit in an experimental setting.

The literature on intrahousehold allocation, which grew out of the tests of the collective model of the household, prompted the recognition that the impacts of interventions differ depending on which member of the household is targeted (Haddad, Hoddinott, & Alderman, 1997). This led to the deliberate targeting of many such interventions to women, such as conditional cash transfer (CCT) programs, even if only observational evidence was available to support the claim that increasing resources controlled by women would increase investments in health and nutrition. In turn, the evaluations of these interventions, many of which involved experimental designs (such as Mexico's PROGRESA), generated evidence that has expanded our understanding of how men and women in rural households make decisions. Eventually, the findings from the impact evaluations led to closer scrutiny of the assumptions on which such programs were based, particularly those related to household decision making and the degree to which household decisions were made jointly, rather than independently.

We begin by asking what has been learned from household models since Becker and Boserup and use this to frame more recent findings about household behavior from three types of analyses: observational studies, experimental games, and impact evaluations. We conclude by pointing out implications for data collection, identifying methodological work to continue examining the assumptions that underlie economists’ approaches to this topic, and suggesting a broader research agenda on social norms and jointness that draws on social science disciplines beyond economics.

## 2 WHAT HAVE WE LEARNED FROM HOUSEHOLD MODELS?

Theoretical models of household behavior are useful for generating hypotheses that can be tested empirically. They can also be used to predict the responses of households and individuals to policy changes. Since the 1980s, the dominant model of household behavior has been the unitary model, which conceptualizes households as groups of individuals who have the same preferences and fully pool their resources (Becker, 1981). The agricultural household model is a version of a unitary model, one which includes both production and consumption decisions (Singh, Squire, & Strauss, 1986). Accumulating empirical evidence has shifted this concept of the household in which households decide “as one” to a “collective” model in which individual household members may have different preferences, may not completely pool resources, and may bargain over outcomes in both production and consumption (Alderman, Chiappori, Haddad, Hoddinott, & Kanbur, 1995).

Because there are many reviews of theories of household behavior (Fafchamps & Quisumbing, 2007; Schultz, 2003; Strauss & Thomas, 1995), we do not attempt an exhaustive review; rather, we focus on what we have learned from the paradigm shift to the collective model of the household. Much of the empirical evidence supporting the collective model of the household comes from data on rural households in developing countries. These have included studies showing differential propensities to spend out of income controlled by men or women (e.g., Hoddinott & Haddad, 1995 for Cote d'Ivoire); differential effects of men's and women's assets on consumption (Doss, 2006 in Ghana) and household expenditures and schooling (Quisumbing & Maluccio, 2003 in Bangladesh, Ethiopia, Indonesia, and South Africa), incomplete sharing of risk within the household (Doss, 2001; Goldstein & Udry, 2008; both in Ghana; Dercon & Krishnan, 2000 in Ethiopia); and inefficiency of resource allocation between plots managed by men and women (Udry, 1996 in Burkina Faso). These findings influenced the design of programs that targeted women, such as CCT programs, which have in turn generated a large pool of evidence that resources controlled by women are associated with investments in child schooling, health, and nutrition (see the systematic review by Yoong, Rabinovich, & Diepeveen, 2012).

All collective models of the household have two common features: they allow different decision makers to have different preferences and do not assume a single household welfare index or utility function (Chiappori, 1992). Much of this literature uses a cooperative bargaining model that is rooted in game theory. These studies begin with the assumption that a household will reach an efficient outcome. Thus, a household could not produce more, simply by reallocating labor or other resources, and goods and services could not be reallocated across household members to make at least one better‐off without making anyone worse‐off. Assumptions that preferences differ by gender then allow tests of how men's and women's bargaining power affects outcomes.

Attention to individuals’ outside options or “threat points” is an important contribution of cooperative bargaining models. The outside option is the amount of resources each individual could access if they were not part of the household, and thus each individual must obtain at least this amount within the household or they will leave. Depending on the context, “leaving” could involve divorce or desertion, or it could involve simply opting out of pooling resources and making joint decisions (“separate spheres”). The important policy insight from the collective model is that outside options affect household resource allocation. For example, women's wages, whether in formal employment or in public works programs, affect household resource allocation, even in households where women are not employed. Changes in laws governing men's and women's property rights and transfer programs, particularly those targeting specific household members, change the outside options of individuals.

Shifting from the unitary to the collective model of the household has expanded our understanding of household behavior as well as policy options to affect it. Yet, two areas remain unresolved. First, there are clearly documented cases in which households do not reach efficient outcomes. In these situations, noncooperative bargaining models may be more appropriate: they do not assume that resources are pooled and explicitly allow for outcomes where potential gains from cooperation have not been realized. Individuals may act strategically; each individual makes separate but interrelated production and consumption decisions based on his or her own preferences and interests and expectations of what others will do. These models provide a framework to test the efficiency assumptions, and several studies, particularly those on agricultural production, find outcomes that are consistent with noncooperative bargaining models, but not with collective models.

For example, a now classic study is that of Udry (1996), who found that total household crop yields could have been increased by shifting fertilizer from men's fields to women's fields. McPeak and Doss (2006) found that male household heads among East African pastoralists located the household farther from town to limit women's milk marketing. Experimental games have identified strategic behavior between spouses (Ashraf, 2009), observed couples making choices that do not maximize surplus (Iversen, Jackson, Kebede, & Munro, 2011), and concluded that outcomes differ depending on the process through which income was acquired (Dasgupta & Mani, 2015).

Second, the collective approach, which has been strongly influenced by bargaining models, often misses key elements of household dynamics. They emphasize competition and rivalry among household members. Yet, even if household members do not completely pool resources, the fact that people form households, share ownership and control over some resources, work together on family farms, produce some output jointly, have and raise children together, and share in some consumption indicates that there are gains to jointness in gender and family dynamics (Doss & Meinzen‐Dick, 2015; Fafchamps & Quisumbing, 2007). Understanding both individual roles within households and the levels of cooperation, including joint decision making and ownership of resources, is essential to analysis of households, especially in rural areas where households engage in both production and consumption. Jointness and cooperation—and possible gains to efficiency and welfare from increasing cooperation—has until recently either been assumed or has been neglected in the analysis of household behavior.

## 3 KEY FINDINGS FROM OBSERVATIONAL STUDIES

Innovations in data collection, particularly the collection of sex‐disaggregated data, have allowed for new insights into a range of issues about rural households; here, we highlight three.

### 3.1 Productivity

An extensive literature documents the gender gaps in agricultural productivity. With the increased availability of plot‐level data that identify owners and managers, such as the World Bank's Living Standards Measurement Study‐Integrated Surveys on Agriculture (LSMS‐ISA), these analyses often compare yields, profits, or the value of output on men's plots and women's plots. Most of these studies find significant gender gaps in favor of men (Aguilar, Carranza, Goldstein, Kilic, & Oseni, 2015; de Brauw, 2015; Oseni, Corral, Goldstein, & Winters, 2015; Slavchevska, 2015). These studies typically compare men's plots and women's plots across the entire data set. They assume that each plot has a single decision maker and that the decisions on each plot are made independently.^1^ They do not explicitly analyze the productivity of different people within the same household.

A smaller literature provides insights into the intrahousehold dynamics of agricultural productivity, considering productivity on plots within the same households. First, households do not necessarily obtain efficient outcomes across plots within the household. Analyzing men's and women's plots within households in Burkina Faso, Udry (1996) finds large gender gaps and concludes that total output for the household could be increased if inputs, particularly fertilizer and men's labor, were shifted from men's plots to women's plots within the same households. Using later rounds of the same panel data, Akresh, Chen, and Moore (2016) find that wives in monogamous households have significantly lower yields relative to the household head than do wives in polygynous households. They attribute this to greater cooperation between cowives than between husbands and wives in polygynous households. A third study in Burkina Faso finds that the differences in yields are primarily between the household head and others in the household, whether the others are men or women. Men who are not the head obtain similar yields to women (Kazianga & Wahhaj, 2013). These findings suggest that there is scope for increased cooperation to increase productivity.

Both gender and marital status play a role in intrahousehold agricultural productivity. In Ghana, Goldstein and Udry (2008) find large gender gaps in profits from plots farmed by husbands and wives. They attribute the difference to longer fallow periods on plots farmed by men than on plots farmed by women. Aguilar et al. (2015) do not specifically look at intrahousehold issues, but do find that most of the productivity gap in Ethiopia is driven by large differences in productivity between men and unmarried women. Married women do not have the same disadvantage, although there are relatively few married women plot managers in their sample.

A second issue that arises is the need to consider both individual and jointly managed plots. Some of the analyses simply drop jointly managed plots and only consider those managed solely by a man or woman, but even in West Africa, where separate men's and women's plots are common, some plots are reported as owned or managed jointly (Doss, Kovarik, Peterman, Quisumbing, & van den Bold, 2015). The evidence suggests that there are some productivity differences across individually and jointly managed plots (De la O Campos, Covarrubias, & Patron, 2016), yet the patterns differ across locations. In Kenya, plots managed by women tended to use less fertilizer and less labor than either plots managed by men or those managed jointly (Diiro, Seymour, Kassie, Muricho, & Muriithi, 2018). Among polygynous households in Malawi, jointly managed plots in these households have higher yields and higher crop values than either men's plots in polygynous households, or plots in monogamous households (Damon & McCarthy, 2019).

These plot‐level analyses all implicitly assume that the decisions about each plot are made independently, yet that would be very surprising were it to be true. Even when people farm individual plots, they are doing so within the context of living in a household and sharing some resources and decisions. Choices about which crops to grow, which inputs to use, and how to allocate one's labor among the various plots and other activities, both paid and unpaid, are made within the context of what others in the household are doing.

Data on the empowerment of individuals within agricultural households provide additional insights. In particular, the availability of data on empowerment for both male and female primary adults in the same household, collected to compute the Women's Empowerment in Agriculture Index (WEAI) (Alkire et al., 2013), allows the comparison of empowerment gaps within households. Taking advantage of nationally representative data in Bangladesh, Seymour (2017) estimates a stochastic frontier production function that includes a measure of women's empowerment. Although the relationship between the WEAI score and technical efficiency is weak, the relationship between the empowerment gap of husbands and wives and technical efficiency is stronger. Technical efficiency is higher when the empowerment gaps are lower. These findings hold for both plots managed only by men and those managed jointly by men and women. (Married women do not manage plots independently in Bangladesh.) This paper suggests that women's relative level of empowerment affects household‐level agricultural productivity, whether or not women are reported as the plot managers.

All of these analyses suggest that we have much to learn about the dynamics of agricultural productivity within households. Analyzing productivity at the plot level ignores the relationships among the plots and their managers, but only considering the household level ignores that individuals within the household may have different levels of influence over particular activities, crops, and plots. The evidence suggests that there may be scope for increased cooperation and productivity among household members in some contexts. But further evidence would be needed on the barriers that limit cooperation within households.

### 3.2 Risk pooling and management

Social norms that influence the importance given to men's, women's, and joint assets may affect households’ risk management strategies. Although the early literature on risk‐pooling showed that household members do not perfectly share risk (Doss, 2001, on Ghana; Duflo & Udry, 2004, on Cote d'Ivoire), more recent studies show that social norms and the extent to which incomes and assets are solely or jointly controlled or managed may affect both the extent of risk‐pooling and households’ welfare outcomes.

There are clear contrasts in the way risk‐smoothing strategies in response to shocks affect men's, women's, and jointly held assets in Bangladesh and Uganda (Quisumbing, Kumar, & Behrman, 2018). Covariate and idiosyncratic shocks have different effects on solely and jointly owned assets and these effects differ by country. During the food price crisis in 2007–2008, jointly held land and assets in Bangladesh were better insured against food price increases compared to jointly held assets and wives’ assets in Uganda. The authors posit that differences in the institution of marriage and cultural concepts of joint and individual ownership affect the extent to which joint or individually owned assets are used to cope with shocks. In Bangladesh, the results show generally insignificant impacts on joint land and asset holdings—while individual assets are sacrificed at the margins—indicating that husbands and wives try not to sell assets that are the economic base of the household unit. In contrast, in Uganda, husbands’ assets appear better insured than wives’ or even joint assets.

The importance of social norms and the extent to which men and women pool resources within the household in mediating the impacts of shocks is highlighted strongly in two studies on Malawi. Asfaw and Maggio (2017) examine how social norms regarding land inheritance tenure interact with joint or sole management of land in mediating the impact of weather shocks on household welfare outcomes. They find that temperature shocks adversely affect household consumption, food consumption, and caloric intake, but rainfall shocks do not have consistent impacts. When women solely manage household plots, temperature shocks not only affect the above welfare measures, but also impinge on nonfood consumption. These effects are not found in households where men solely or jointly cultivate land, suggesting that households where women are the sole managers of land are more vulnerable. They also find that in matrilineal districts, where women traditionally have more secure property rights, households where women are the sole managers of land are less vulnerable to shocks compared to similar households living in patrilineal districts.

Assumptions about the extent of income pooling also affect our understanding of risk‐pooling. Josephson (2017) tests the assumption that all household income is pooled, accounting not only for income earned individually by men and women, but also joint income. Although she rejects the hypothesis of complete income pooling and full insurance within the household, she finds evidence that household members partially insure one another for expenditure on essential goods (including food, clothing, education, and healthcare) but not for luxury goods (such as cigarettes and alcohol, recreation, and housing and utilities). This result holds only when joint income is included; when only individual incomes are considered, her findings show that household members do not pool income or insure against shocks. This suggests that omitting joint relationships fails to account for an important dynamic in household analysis. Similar to Asfaw and Maggio (2017), Josephson also examines the implications of living in a patrilineal or a matrilineal area on risk‐pooling. She finds that households in matrilineal societies completely pool income and fully insure one another, whereas households in nonmatrilineal societies do not. This difference is not driven by the sex of the household head, suggesting that social norms matter.

Recent analyses on risks and shocks are made possible by the increased availability of geo‐referenced data on weather‐related variables, such as temperature and rainfall, on a sufficiently disaggregated scale that allows mapping to household surveys. The growth in the availability of “big data” will provide opportunities to examine how conditions at the macro‐ and meso‐levels affect outcomes within the household, if the appropriate identifiers are available to link data sources.

Together, these studies suggest that we should be analyzing the conditions under which household members pool their risk. The research presented here suggests that social norms and the extent to which household members pool income and other resources will affect how they handle shocks.

### 3.3 Spousal agreement

In addition to increased data at the plot level, a number of surveys have interviewed multiple people within the household. This provides additional new insights into the dynamics of agricultural households. One challenge has been to understand when different responses from husbands and wives provide new information about household dynamics and how best to analyze this information.

Increasing evidence demonstrates that when you ask multiple people within the household the same questions, you get different answers. This has been found for reports of labor force statistics in Tanzania (Bardasi, Beegle, Dillon, & Serneels, 2011), housing values in Ecuador, Ghana, and Karnataka, India (Doss et al., 2018), income in Malawi (Fisher, Reimer, & Carr, 2010), financial services in Paraguay (Fletschner & Mesbah, 2011), assets and decision making in Bangladesh (Ambler, Doss, Kieran, & Passarelli, forthcoming), and assets in Uganda (Kilic & Moylan, 2016), among others. These studies all find that not only do responses differ, but they also differ systematically by gender.

Several analyses provide insights specifically into decision making in agricultural households. Using Tanzanian survey data where both husbands and wives were interviewed, Anderson, Reynolds, and Kay (2017) find extensive differences in responses regarding whether women are involved in household and farming decisions, depending on both who responds and on the specific decision considered. Husbands and wives do not necessarily provide the same responses and their responses are not consistent across decisions. Similarly, Twyman, Useche, and Deere (2015) find different responses from husbands and wives about land ownership and agricultural decision making. In this sample in Ecuador, men report lower levels of involvement of women in agricultural decision making than do women. Similarly, Kosec, Akramov, Mirkasimov, and Song (2018) find different responses from husbands and wives regarding women's participation in major economic decisions, financial management decisions, and nonfinancial decisions like negotiations with neighbors—with wives more likely than husbands to say that wives participate in decision making.

The systematic differences in responses between husbands and wives cannot be attributed simply to measurement error, but we are interested not only in whether they provide different responses, but also how their responses are associated with outcomes—either in terms of agricultural decisions or welfare outcomes—for the household. Analyzing the relationship between responses and autonomy in Ghana and Bangladesh, Seymour and Peterman (2018) conclude that the level of agreement on decision making is indicative of the underlying power dynamics within households. Ambler et al. (forthcoming), also using data on Bangladesh, argue that the different responses from husbands and wives on asset ownership and household decision making are consistent with a story of asymmetric information within the household.

Most of these studies use observational data where both husbands and wives are interviewed. Two other papers randomly assign households to survey arms where different household members are interviewed. One study in Ecuador uses three approaches: interview only the husband, interview only the wife, and interview both husband and wife separately, with each knowing that the other will be interviewed (Alwang, Larochelle, & Barrera, 2017). They find that men and women have very different responses regarding their responsibilities for decisions on pest and farm management. Men tend to claim sole responsibility, while typical responses from women include both sole and joint responsibility. When husbands and wives are both interviewed, the responses are more likely to be that the decision making is joint. An analysis of gendered patterns of asset ownership using a more complex survey design finds that the patterns of individual asset ownership reported depend both on who the respondent is and whether others within the household are also interviewed (Kilic & Moylan, 2016).

It is useful to note that much of the work on intrahousehold bargaining uses survey responses reported by one household member. Various proxies for bargaining power are used in the literature, including differences in husbands’ and wives’ education, land or other asset ownership, unearned income, and so on (see Doss, 2013, for a discussion of these proxies). Typically, survey data have one report on these from one household member. Thus, it is not necessary to interview multiple people within the household to do intrahousehold analyses, but the recent evidence on disparities in response suggests that the findings may differ depending on who is interviewed.

Again, the recent work has raised a number of new questions. When is it appropriate to interview only one person and rely on their answers? And when is it necessary, despite the additional expense, to interview multiple members of the household? Clearly, if we want to know someone's preferences or knowledge, we should not rely on a proxy respondent. Further work is also needed to identify when useful information is embedded in the fact that spouses provide different answers and when it is simply measurement error.

## 4 EVIDENCE FROM EXPERIMENTS

One methodological innovation used to gain an additional perspective on household cooperation has been lab‐in‐the‐field experiments, particularly public goods games. Public goods games are those in which each player is given an amount of money which they can keep or contribute to put in the “public” or household pot. The money that is put in the household pot is then increased (often multiplied by 1.5) and then divided among the players. The *highest* payoff to the household overall occurs if both players put all of their money into the household pot. If, however, one player does not expect the other to contribute to the pot, then their best response is not to contribute.

A number of studies find inefficiencies when considering the contributions of husbands and wives. In the simple game where the household pot is evenly split, spouses generally do not contribute their entire allocation to the household pot, potentially leaving some income on the table (Barr, Dekker, Janssens, Kebede, & Kramer, 2019; Fombe, Sama‐Lang, Fonjong, & Mbah‐Fongkimeh, 2013; Iversen et al., 2011; Munro, Kebede, & Tarazona‐Gomez, 2014).^2^ This does not imply that households are *never* fully cooperative in this sense; instead only that, on average, households do not *fully* cooperate.

A number of variations on the game provide further insights. Although Iversen et al. (2011) reject the notion that spouses maximize the surplus, they find significant differences across villages in Uganda, suggesting that the patterns are heterogeneous. One variation is to alter the allocation rule. They have three options in the standard approach, the surplus is split evenly, but they also introduce an option whereby the control over how to split the surplus is given either to the husband or the wife. They find that when women control the allocation, both husbands and wives contribute more to the household pot. However, they do not find evidence that women generally contribute more than men.

Kebede, Tarazona, Munro, and Verschoor (2013) also introduce variations where the allocation decision may be given to the husband or wife. In addition, the authors ask spouses about what they expect the other to contribute. Although husbands’ expectations of their wives’ contributions are higher than their wives’ actual contributions, wives’ expectations of what their husbands will contribute are lower than what the husbands do contribute. Although overall men contribute slightly more than women, on average, people contribute only about half of their allocation to the common pot, leaving substantial surplus unrealized.

Two papers analyze whether the patterns seen in the lab‐in‐the‐field games are correlated with actual agricultural outcomes. Lecouter and Jassogne (2019) use a public goods game in combination with an elicitation of consumption preferences regarding the household pot. They find that households that are both more cooperative and egalitarian in their consumption (neither partner claiming more than their share) are more likely to invest in sustainable intensification of food and cash crops. Analyzing pastoralist households in Northern Senegal, Hoel, Hidrobo, Bernard, and Ashour (2017) demonstrate that households that obtain less cooperative outcomes in the public goods games are the ones largely driving the gender gap in milk production. In these households, cows owned by women produce 10.6% less milk than those owned by men, even after controlling for household, owner, and cow characteristics.

The findings from these experiments suggest that there may be gains to increasing cooperation within the household. Yet, it is unclear whether increased cooperation would work outside a study setting, and what interventions would increase cooperation. It is also unknown whether there may be unintended consequences of cooperation, for example, if cooperation is used to mask disagreements or suppress individual agency. Findings from impact evaluations, discussed below, provide promising answers to some of these questions.

## 5 EVIDENCE FROM IMPACT EVALUATIONS

How would understanding household behavior help us design and implement more effective interventions to achieve desired development outcomes? The earlier literature on intrahousehold allocation, based mostly on observational studies (reviewed in Quisumbing, 2003 and Yoong et al., 2012), suggested that increasing women's bargaining power—often by increasing their outside options by increasing resources under their control—would result in better development outcomes, such as improved child health and nutrition. Based on these findings, many development programs and interventions targeted both cash and in‐kind transfers to women (Baird, Ferreira, Özler, & Woolcock, 2014); however, because they impose conditionalities, CCT programs have been criticized (Molyneux, 2006) for reinforcing women's traditional gender roles as caregivers. In many cases, this not only increases women's workload, as the task of attending community meetings and bringing children to clinics often falls on them, but also neglects their roles in the productive sphere. By focusing on men's and women's expected roles in the Beckerian sense, with men as farmers or producers and women as caregivers, they may have unintentionally reinforced social norms that limit women's agency. In addition, by focusing on men's and women's bargaining power instead of potential areas of cooperation, they may unwittingly have missed opportunities to make interventions more effective.

The Beckerian idea that men specialize in the productive sphere and women in the reproductive sphere has also influenced the design of many agriculture‐nutrition programs. Most agricultural programs target extension advice about agriculture to men, and nutrition messages, as relevant, to women. But this approach may have unintended consequences. One example comes from the evaluation of the Reaching End User Orange Sweet Potato project of HarvestPlus in Uganda (Gilligan, Kumar, McNiven, Meenakshi, & Quisumbing, 2014). This project disseminated biofortified orange sweet potato (OSP) vines to farmers’ groups and gave nutrition messages about vitamin A to women, but not to their husbands. In examining adoption decisions within households, Gilligan et al. (2014) found that plots of land exclusively controlled by women are not more likely to contain OSP, but plots under joint control of men and women, in which a woman has primary control over decision making, are significantly more likely to contain OSP. Plots exclusively controlled by men are the least likely to contain OSP. This evidence indicates that women play an important, and often a leading role, in the decision to adopt OSP, but that this decision is often jointly made with their husbands. Because of the jointness of these decisions, the current strategy of targeting only women with nutritional training may be missing an opportunity to create an awareness of the benefits of OSP among men.

Gender norms may also limit the potential impacts of programs that could empower women. BRAC's randomized Challenging the Frontiers of Poverty Reduction‐Targeting the Ultra‐Poor project in Bangladesh provided livestock and training to rural women in “ultra‐poor” households (Roy, Ara, Das, & Quisumbing, 2015). For livestock (the primary assets transferred), the program slightly increases ownership by men but causes much larger increases in sole or joint ownership by women. These increases in women's livestock ownership are associated with some increases in their control over the livestock, including the right to sell cattle, which are perceived as “men's assets” in the local context. However, Roy et al. (2015) also find increases in household ownership of many other assets (not directly transferred by the program), which tend to be solely owned by men. This suggests that proceeds from the asset transfer program, although ostensibly transferred to women, are reinvested in assets that are controlled mostly by men. Moreover, while the program did not change the proportion of women who work, it shifted work from outside to inside the home, because the transferred asset (livestock) needed to be maintained within the homestead; this potentially reduced women's mobility. The program also significantly reduced women's voice in a range of decisions—including control over their own income, purchases for themselves, and decision making for household budgeting. These reductions are consistent with economic models that link individuals’ relative control over resources to their intrahousehold bargaining position. Yet, qualitative work among the women in the program suggests that there were many intangible benefits to women, such as increased confidence and social capital. Moreover, because work opportunities outside the home for poor women are poorly paid and stigmatizing given local norms of female seclusion, most beneficiary women preferred to generate income at home, even if it reduced their mobility and increased their workload. Thus, even if the program reduced their voice in household decisions, women themselves tended to frame the gains in terms of higher social status within the community and the household because of their contributions to the economic condition of the household, rather than in terms of individual gains.

In this context, increases in the welfare of the household or collective were valued more than increases in individual bargaining power, suggesting that focusing only on increasing bargaining power may be a very narrow objective of development interventions. We note, however, that this particular BRAC program did not intentionally aim to empower women nor promote women's asset ownership. Instead, it aimed to build assets of poor households as a whole by providing women with assets that can be maintained at home, a productive enterprise that is compatible with social norms that favor female seclusion.

Another example where social norms limited potential empowerment impacts was Zambia's Child Grant Program, a poverty‐targeted, unconditional cash transfer (UCT) given to mothers or primary caregivers of young children aged 0–5. The mixed methods evaluation consisted of a 4‐year longitudinal clustered‐randomized control trial in three rural districts, complemented by a one‐time data collection involving in‐depth interviews with women and their partners. Bonilla et al. (2017) found that women in beneficiary households made more sole or joint decisions, but impacts translated into relatively modest increases in the number of decision domains in which a woman is involved. The qualitative study found that changes in intrahousehold relationships were limited by entrenched gender norms, which designate men as heads of households and primary decision makers. Nevertheless, women felt that the transfer empowered them financially as they were able to retain control over transfers for household investment and savings for emergencies. Thus, even if the program resulted in beneficial gendered impacts, it failed to transform gender norms. Bonilla et al. (2017) also find that intrahousehold decision making is an imperfect indicator for measuring empowerment. Specifically, even though women often state they make decisions (either solely or jointly), if partners disagree, women's preferences are second to men's. This finding questions the validity of empirical measures that aim to capture the concept of “the ultimate decision maker,” as well as challenging the very basis of the concept itself.

An interesting conclusion from the Zambia impact evaluation sheds light on conditional versus unconditional transfer programs. Conditional programs have been criticized for reinforcing traditional gender norms, owing to women's likelihood of shouldering additional activities related to household welfare (taking children to clinics and attending information sessions) (Molyneaux, 2006). Bonilla et al. (2017) argue that the lack of coresponsibilities in UCTs ensures that women do not incur additional time burdens that could otherwise have been shared with other household members or partners.

Other programs have deliberately included women's empowerment as part of the impact pathway to achieving desired outcomes. One example is Helen Keller International's Enhanced Homestead Food Production in Burkina Faso, which aimed to improve children's nutritional status, through the provision of agricultural assets and delivery of a behavior change communication strategy focused on agricultural activities and optimal infant and young child feeding, health, hygiene, and care practices (Olney, Pedehombga, Ruel, & Dillon, 2015). These program inputs were expected to improve children's nutritional status by increasing women's production of nutrient‐rich foods and children's intake of these foods. The program established community gardens where women could grow vegetables, and enhanced women's income and control over resources through sale of surplus production. By increasing access to program inputs, control over resources, and maternal knowledge of and ability to implement optimal agriculture, nutrition, and health practices, the program was expected to improve women's bargaining power, particularly over the resources needed for maternal and child health and nutrition. A cluster‐randomized controlled trial showed that the program reduced gender asset inequality: women's value of agricultural assets in intervention villages increased, whereas men's decreased (van den Bold et al., 2015). Although the project had no impact on the area of land cultivated by either men or women, qualitative work indicates that gender norms became more favorable toward women's landownership in treatment as compared with control areas. A follow‐up study (Heckert, Olney, & Ruel, 2019) focusing on women's empowerment found that improvements in women's empowerment in the domains of spousal communication, purchasing decisions, healthcare decisions, and family planning decisions contributed to the program's impact on reducing wasting, with the largest share being attributable to spousal communication. Improvements in women's empowerment did not contribute to the increase in hemoglobin.

Recent interventions often involve more deliberate attempts to change gender norms to improve spousal communication and increase cooperation within the household. One such study uses a quasi‐experimental design with qualitative methods to examine the effects of a participatory intervention on gender relations in Malawi (Kerr, Snapp, Chirwa, Shumba, & Msachi, 2007). The Soils, Food and Healthy Communities project integrates sustainable agriculture and nutrition using a participatory approach to improve child nutrition, in which farming households develop and test different strategies, including crop diversification, organic methods, and nutrition education (Preibisch, Herrejon, & Wiggins, 2002). The project team modified the program to include a community‐based participatory nutritional education intervention called “recipe days” to promote healthy feeding of complementary foods for under‐five children, share skills on how to prepare diverse recipes, and act as a platform to encourage more equitable household gender roles. The “recipe days” included not only the preparing and sharing of recipes based on local crops, but also discussions of the role of men in childcare and the household division of labor and decision making, using a “dialogue” approach rather than a “lesson.” The study (Kerr et al., 2007) provided evidence that participatory education supported new concepts of masculinity that encouraged men to be more involved in cooking and child care. The transformational approach, involving grandparents and spouses, a focus on agriculture and food security, as well as involving male community leaders were some of the reasons named by respondents as examples of changed gender norms.

Both the Heckert et al. study and the Kerr et al. study are evaluations of recent nutrition‐sensitive agricultural programs. Because these studies tend to have nutrition as a primary outcome, agricultural economists who focus on productivity or income‐related measures often miss them. Considering a broader definition of desired development outcomes may enhance our understanding of household behavior.

## 6 CONCLUSIONS

We use the theoretical and empirical contributions of Ester Boserup and Gary Becker to frame the contributions of recent research to our understanding of household behavior in rural areas of developing countries. Boserup and Becker highlighted the importance of social norms, which in turn influence gender roles, but recent empirical work shows that while these norms exist, they are changeable and changing. The demographic transition, processes of structural transformation, the shift from agriculture to nonagriculture, urbanization, and globalization underlie these changes in gender norms and individual agency. Becker's prediction that individuals in households will specialize based on social norms does not adequately recognize that both men and women are involved in productive and reproductive work and on decisions on those spheres, and that these decisions may be made primarily by one person or they may be made jointly. It is not only important to understand whether these decisions are made solely or jointly, but also the extent to which individuals exercise agency or choice to make those decisions.

Empirical evidence from tests comparing the unitary and collective models of household behavior has convincingly led to the rejection of the unitary model. However, its focus on bargaining models has also led to the neglect of cooperation within the household. Rather than assume that all households cooperate, or demonstrate that they do not, it would be interesting to find out why some households cooperate and others do not, and the consequences thereof. Lab‐in‐the field experiments may help us understand patterns of spousal decision making, but these small experiments need to be carefully interpreted within the contexts of the societies in which these studies are conducted. Contributions from sociology and anthropology as well as qualitative work on household decision making will be important in advancing our understanding of rural household behavior. Researchers who study household behavior also need to be aware of their assumptions, which are often culturally grounded, and to challenge and test them. Methodological innovations in data collection and study design provide more scope for testing our assumptions about individual and household behavior in different contexts.

The literature of the last 40 years has also challenged the notion that we can treat agricultural households as though they are profit‐maximizing firms. The evidence is now convincing that often households do not obtain all of the potential surplus from their farms. This may be due to social norms that affect the roles and responsibilities of various individuals within the household, and individuals, at times, may seek their own gain and control over resources, at the expense of overall household production.

Many of the studies that we review are possible only because of the increased availability of data on individuals as well as resources controlled by individuals within households. Such data, like the LSMS‐ISA and surveys collecting the WEAI, allow us a deeper look into rural household behavior. Linking these data to spatial or georeferenced data, as well as big data, would help us to understand better the broader context within which household decisions are made. Expanding our definition of outcomes of interest beyond those traditionally studied in agricultural economics—such as human capital, encompassing education, health, and nutrition—as well as outcomes directly related to well‐being would expand our scope of understanding household behavior as well. Yet, simply having more sex‐disaggregated data and data on smaller units of production, such as plots, does not always lead to more fruitful analyses. For example, analyzing productivity only at the plot level ignores the relationships among the plots and their managers.

Finally, while our review of the literature has focused on data and methods, we emphasize that assumptions about individual and household behavior can be very influential in the design of policies and interventions. We would do well to continually question our assumptions about household behavior when we design and implement development interventions, systematically evaluate the impacts of these interventions, and revise our models if the results of these evaluations are contrary to what we expect.

## Footnotes

1 See Doss (2017) for an extensive discussion of issues around gender and agricultural productivity.

2 Castilla (2015) finds similar results in India for a variant on this game in which one spouse is given an allocation and allowed to transfer any or all of it to their spouse. The spouse receives triple the amount transferred and may then transfer any or all of it back.
